# Minocycline in acute stroke: A systematic review and meta-analysis of randomized controlled trials and trial sequential analysis

**DOI:** 10.1097/MD.0000000000051287

**Published:** 2026-10-23

**Authors:** Muhammad Shahzaib, Muhammad Roshaan, Gaaitri Lohano, Marium Fawad, Haram Nabeel, Muhammad Noraiz Abbas Khan, Muhammad Rehan Zahid, Muhammad Nabeel, Nimra Afzal, Muhammad Shahryar, Eman Shahzad, Prince Kusimw’iragi

**Affiliations:** a King Edward Medical University, Lahore, Pakistan; b Liaquat University of Medical and Health Sciences, Jamshoro, Pakistan; c Dow University of Health Sciences, Karachi, Pakistan; d Sialkot Medical College, Sialkot, Pakistan; e DHQ Teaching Hospital, Dera Ismail Khan, Pakistan; f Khawaja Muhammad Safdar Medical College, Sialkot, Pakistan; g Université Evangélique en Afrique (UEA), Bukavu, Democratic Republic of Congo.

**Keywords:** acute ischemic stroke, acute stroke, intracerebral hemorrhage, meta-analysis, minocycline, neuroprotection

## Abstract

**Background:**

Minocycline has emerged as a potential adjunctive neuroprotective therapy in acute stroke owing to its anti-inflammatory, antiapoptotic, and blood–brain barrier-stabilizing effects. We aimed to evaluate the efficacy and selected safety-related outcomes of minocycline in acute stroke, including ischemic and hemorrhagic subtypes.

**Methods:**

This systematic review and meta-analysis followed Preferred Reporting Items for Systematic Reviews and Meta-Analyses guidelines. Major databases were searched through March 20, 2026 for randomized controlled trials comparing minocycline with control or standard care in acute stroke. Risk ratios (RRs) and mean differences were pooled with 95% confidence intervals (CIs). Risk of bias was assessed using ROB 2, certainty of evidence using Grading of Recommendations Assessment, Development, and Evaluation, and robustness using trial sequential analysis.

**Results:**

Nine trials including 2186 participants were analyzed. Minocycline significantly reduced 3-month National Institutes of Health Stroke Scale scores in acute ischemic stroke (mean difference −2.43, 95% CI −4.42–−0.44; *P* = .0169), but not in intracerebral hemorrhage. Functional independence at 3 months showed a favorable signal in ischemic stroke (modified rankin scale 0 to 2: risk ratio 1.40, 95% CI 1.05–1.86; *P* = .0219), while favorable outcome defined as modified rankin scale 0 to 1 was not significantly improved. Barthel Index also favored minocycline in ischemic stroke, although overall results were not statistically significant. Mortality, recurrent vascular events, hemorrhagic transformation, hematoma volume, perihematomal edema, and Matrix Metalloproteinase-9levels were not significantly altered. Trial sequential analysis indicated insufficient evidence for definitive conclusions.

**Conclusion:**

Minocycline may improve neurological recovery in acute ischemic stroke; however, these findings remain exploratory because of substantial heterogeneity, low-certainty evidence, and inconclusive trial sequential analysis. Larger standardized trials are required.

## 1. Introduction

Stroke is the leading cause of death with chronic impairment worldwide, with a particularly high impact in low- and middle-income countries.^[1]^ Despite improvements in acute stroke management, including thrombolytic therapy and endovascular interventions, many patients remain unentitled due to narrow therapeutic windows or contraindications, spotlighting the urgency for adjunctive neuroprotective therapies that can boost outcomes across diverse patient populations.^[1,2]^

Minocycline, a second-generation semisynthetic tetracycline, has emerged as a potential neuroprotective agent. Its lipophilic properties allow it to efficiently cross the blood–brain barrier, where it produces diverse outcomes, including inhibition of microglial activation, suppression of inflammatory cytokines, reduction of matrix metalloproteinase activity, stabilization of the blood–brain barrier, and attenuation of apoptosis.^[3–6]^ Preclinical development invariably proves that minocycline reduces infarct size, limits peri-hematomal edema, and mitigates neuronal loss in both ischemic and hemorrhagic stroke models.^[4,5,7]^ Moreover, minocycline does not compromise the fibrinolytic activity of intravenous tissue-plasminogen activator, contributing to the likelihood of extending the therapeutic window for reperfusion therapy in acute ischemic stroke (AIS).^[8,9]^ Clinical documentation, however, remains restricted and diverse. Early-phase randomized controlled trials (RCTs) investigating minocycline in AIS and intracerebral hemorrhage (ICH) report variable results for neurological improvement and functional recovery.^[10–12]^ The reason for these inconsistencies is differences in dosing, timing of administration, route of delivery, and patient selection. Moreover, the tolerability profile of minocycline, including hepatotoxicity, vestibular effects, and tolerability in older populations, needs additional assessment.^[13]^

Previous systematic reviews and meta-analyses have proposed that minocycline may optimize neurological outcomes and functional recovery after stroke, but these analyses were constrained by small sample sizes, limited trials, and inadequate subgroup analyses. ^[5]^ Since then, many new RCTs have been published, providing supporting information on both efficacy and safety across stroke subtypes. So far, the repercussions of minocycline on clinically productive outcomes, including mortality, modified Rankin Scale (mRS) scores, NIH Stroke Scale scores, hematoma volume, and peri-hematomal edema, need to be systematically analyzed.

The main objectives of this study are to integrate evidence from recent RCTs to assess efficacy and safety outcomes across different stroke subtypes, explore heterogeneity related to dosing and timing of administration, and provide a unified assessment of minocycline’s potential as a neuroprotective agent in acute stroke management.

## 2. Methods

### 2.1. Protocol and registration

This systematic review and meta-analysis followed the Preferred Reporting Items for Systematic Reviews and Meta-Analyses 2020 guidelines.^[14]^ Our review protocol was also prospectively registered with the International Prospective Register of Systematic Reviews with the assigned registration number (CRD420261373320). Ethical approval and informed consent were not required because this systematic review and meta-analysis used previously published aggregate data and involved no direct participant recruitment or identifiable individual-level data.

### 2.2. Literature search

A systematic literature search was conducted across PubMed, Embase, Cochrane Central, and ScienceDirect from inception to March 20, 2026. The search strategy employed a combination of controlled vocabulary and free-text terms, including Medical Subject Headings (MeSH) terms and relevant free-text keywords related to “Stroke,” “Cerebrovascular Disease,” “Cerebral Ischemia,” “Hemorrhagic Stroke,” “ICH,” “Neuroprotection,” “Minocycline,” and “Minocin.” The search strategy incorporated Boolean operators (“AND” and “OR”) to enhance search precision. Reference lists of included articles and pertinent reviews were manually searched to capture additional relevant studies. A detailed search strategy is given in Supplementary Table 1, Supplemental Digital Content 1.

### 2.3. Study selection and data extraction

After removing duplicates using EndNote, two investigators (R.Z. and H.N.) independently screened titles and abstracts using Rayyan, resolving discrepancies. Subsequently, M.R. and M.S. independently screened full texts according to inclusion criteria; any disagreements were resolved through discussion between the authors. The analysis included RCTs of patients with acute stroke that compared minocycline with placebo or standard care. Eligible studies were required to use randomized allocation; blinding was not required for eligibility. Open-label randomized trials were therefore eligible, with lack of blinding incorporated into the risk-of-bias assessment. Placebo-controlled and standard-care-controlled trials were pooled because both evaluated the incremental effect of minocycline in addition to background acute-stroke management; control type was nevertheless considered a source of clinical heterogeneity.

Data extraction was done independently by two investigators (R.Z. and G.L.) using a standardized data extraction sheet, including the name of the study, first author, and year of publication, mean age, sex distribution, trial name, total number of study participants, prevalence of cerebrovascular risk factors, stroke clinical syndrome, clinical outcomes, and follow-up duration. The number of events and the sample size were extracted for binary outcomes, and mean, standard deviation, and median with 95% confidence intervals (CI) for continuous outcomes. A meta-conversion tool was used to convert the values to the desired units, helping with consistent analysis across studies.^[15]^

### 2.4. Risk of bias and certainty of evidence assessment

The Cochrane risk-of-bias tool for randomized trials (ROB 2) was used to assess the risk of bias by two investigators (M.S. and M.R.). It includes 5 domains: bias due to the randomization process, deviations from intended interventions, missing outcome data, measurement of outcomes, and selection of the reported result and “overall risk of bias” judgment.^[16]^ Funnel plots were generated for descriptive assessment; however, because fewer than 10 studies contributed to individual outcomes, funnel-plot asymmetry was not considered a reliable test of publication bias. Furthermore, the Grading of Recommendations Assessment, Development, and Evaluation (GRADE) framework was used to assess the certainty of evidence.^[17]^ Any conflict was resolved through discussion between the authors.

### 2.5. Statistical analysis

The analysis combined results from multiple studies using either risk ratio RRs (for dichotomous outcomes) or mean difference (MD) (for continuous outcomes), both with 95% CIs. Given the anticipated clinical and methodological heterogeneity across stroke subtype, treatment timing, dose, route of administration, and trial design, a random-effects model was prespecified and applied consistently to all pooled analyses. Statistical heterogeneity was quantified using *I^2^* and assessed using the *χ^2^* test. Heterogeneity was interpreted according to the Cochrane Handbook,^[18]^ with an *I^2^* value of 0% to 40% indicating low heterogeneity, 30% to 60% signifying moderate heterogeneity, 50% to 90% representing substantial heterogeneity, and 75% to100% highlighting significant heterogeneity. A Chi-square test *P*-value below .05 was considered statistically significant for heterogeneity. Sensitivity analysis was employed to determine the source of heterogeneity where present.^[19]^ Trial sequential analysis (TSA) was performed using the RTSA package to assess the conclusiveness of evidence and adjust for random errors.^[20]^ Binary outcome data (events and sample sizes for minocycline and control groups) were entered into a structured data frame. TSA was conducted with a two-sided *α* = 0.05, power = 80%, and a predefined minimal clinically important relative risk of 0.8 (Relative Risk Reduction = 20%) for binary outcomes such as 3-month functional independence mRS (0–2) and mortality. The minimal clinically important difference for 3 months Barthel Index (BI) was 10; for 3-month National Institutes of Health Stroke Scale (NIHSS), it was −2. The cumulative Z-curve was evaluated against conventional and TSA-adjusted boundaries to determine statistical significance. Treatment-initiation windows varied substantially across trials, ranging from early administration to initiation beyond 48 hours after symptom onset. Studies were pooled because all evaluated minocycline during the acute stroke period; however, variation in treatment timing was considered an important source of clinical heterogeneity and was taken into account when interpreting the pooled estimates.

### 2.6. Outcomes

Primary outcomes: mRS, BI, and NIH Stroke Scale NIHSS scores were studied at baseline, day 5 to 7, 1-month, and 3-month time periods. We further dichotomized the clinical outcomes: mRS 0 to1 (favorable functional outcome), mRS 0 to 2 (functional independence), and mRS 6 (mortality).

Secondary outcomes included selected safety-related and neuroprotective outcomes among patients with AIS and ICH. We additionally assessed neuroimaging and biochemical endpoints in ICH patients by analyzing the reduction of matrix metalloproteinase (MMP)-9 levels, peri-hematomal edema, and hematoma volumes. For MMP-9, we additionally extracted the biological sample, analytical assay, measurement time point, and reported metric to assess methodological comparability between trials.

## 3. Results

### 3.1. Baselines and study characteristics

The database search yielded 1269 records. After removal of 423 duplicates, 846 records were screened by title and abstract, of which 826 were excluded. Twenty full-text articles were assessed for eligibility; eleven were excluded due to reasons mentioned in the Preferred Reporting Items for Systematic Reviews and Meta-Analyses flow diagram. Ultimately, 9 RCTs met the inclusion criteria and were included in the qualitative synthesis and quantitative meta-analysis (Fig. 1).

**Figure 1.**
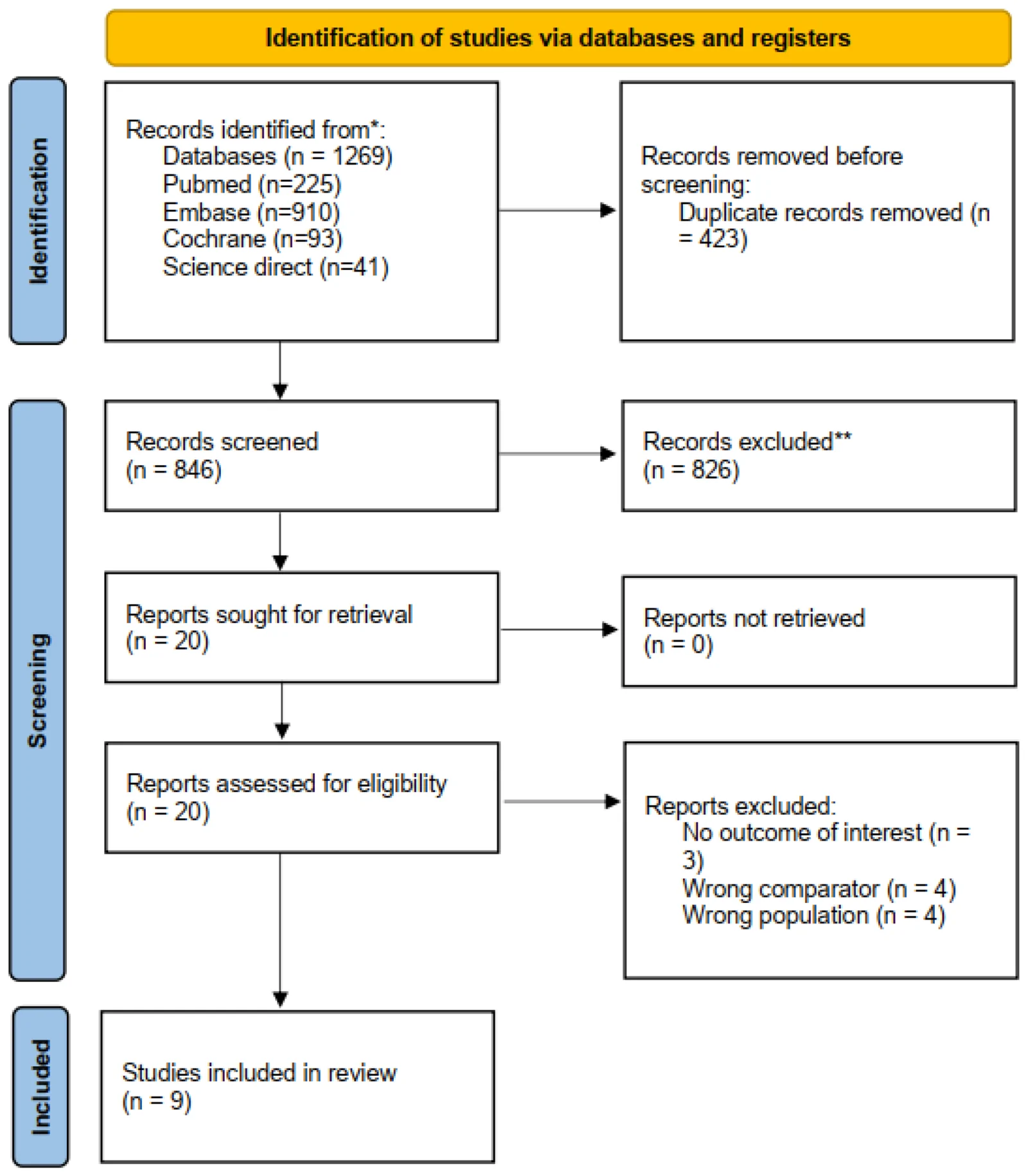
PRISMA study selection flowchart. PRISMA = preferred reporting items for systematic reviews and meta-analyses.

A total of 2186 participants were included across the analyzed trials, with 1086 patients in the minocycline group and 1100 patients in the control group. The studies were conducted across multiple regions, including Australia, Asia, North America, Iran, and China, reflecting a geographically diverse population. Both groups were male predominant, with male populations exceeding 50% of the total included participants in most of the studies. Baseline characteristics of the included population are summarized in Tables 1–2.

**Table 1:** Characteristics of included studies.

Study	Country	No of participants		Patient cohort AIS/ICH		Dosage		Route of administration		Minocycline administration from symptom onset, h	
		Minocycline	Control	Minocycline	Control	Minocycline	Control	Minocycline	Control	Minocycline	Control
Blacker 2013	Australia	21	23	AIS = 21	AIS = 23	Minocycline200 mg, twice daily	Placebo	IV	IV	<6 h	<6 h
Lampl 2007	Asia	74	77	AIS = 74	AIS = 77	Minocycline 200 mg, once daily	Placebo	Oral	Oral	6–24 h	6–24 h
Srivastava 2012	Asia	23	27	AIS = 23	AIS = 27	Minocycline 200 mg, once daily	Vitamin B capsules (placebo control)	Oral	Oral	6–24 h	6–24 h
Chang 2017	North America	10	10	ICH = 10	ICH = 10	Minocycline 10 mg/kg (max 700 mg/d), once daily	Placebo	IV	IV	< 12 h	< 12 h
Fouda 2017	North America	8	8	ICH = 8	ICH = 8	Minocycline 400 mg, once daily	Placebo	IV (day 1) + Oral (day 2–5)	IV (d 1) + Oral (d 2–5)	<24 h	<24 h
Kohler 2013	Australia	44	48	AIS + ICH = 44	AIS + ICH = 48	Minocycline 100 mg, twice daily	No minocycline; routine stroke care	IV	IV	< 24 h	< 24 h
Amiri-Nikpour 2015	Asia	26	27	AIS = 26	AIS = 27	Minocycline 200 mg, once daily	No minocycline; aspirin 100 mg/d in both groups.	Oral	Oral	6–24 h	6–24 h
Shamsaei 2017	Iran	18	18	AIS = 18	AIS = 18	Minocycline 200 mg/day for 5 d	Placebo	Oral	Oral	NR	NR
Lu 2026	China	862	862	AIS = 862	AIS = 862	Minocycline, loading dose = 200mg within 30 min post randomization; maintenance dose = 100 mg/12 hr for 4 d	Placebo	Oral/ feeding tube	Oral/ feeding tube	≤24 h: 20.8 >24–48 h: 43.5 >48 h: 35.7	≤24 h: 20.3 >24–48 h: 45.9 >48 h: 33.8

AIS = acute ischemic stroke, ICH = intracerebral hemorrhage, IV = Intravenous, NR = not reported.

**Table 2:** Baseline characteristics of included patients.

Study	Age mean (sd)		Male (%)		Hypertension (%)		Diabetes Mellitus (%)		Hyperlipidemia (%)		Smoker (%)		Prior AIS/TIA (%)		Prior heart disease (%)		Follow up duration	
	Minocycline	Control	Minocycline	Control	Minocycline	Control	Minocycline	Control	Minocycline	Control	Minocycline	Control	Minocycline	Control	Minocycline	Control	Minocycline	Control
Blacker 2013	62.8 (10.9)	62 (11.5)	65.21	60.86	47.82	65.21	13.04	30.43	52.17	52.17	47.82	47.82	13.04	4.34	21.73	21.73	3 mo	3 mo
Lampl 2007	67.2 (11.1)	66.2 (11.1)	63.51	66.23	60.81	64.93	36.36	25	24.32	35.06	27.02	31.16	22.97	22.07	29.72	31.16	3 mo	3 mo
Srivastava 2012	52.7 (15.3)	57 (14.2)	56.52	66.67	65.21	70.37	17.39	17.39	43.47	44.44	30.43	13.81	NR	NR	26.08	37.03	3 mo	3 mo
Chang 2017	59 (13)	55 (13)	60	50	80	100	20	40	60	70	10	10	10	10	30	10	3 mo	3 mo
Fouda 2017	62 (14.6)	61.8 (11.5)	37.5	12.5	87.5	100	25	50	NR	NR	NR	NR	NR	NR	NR	NR	3 mo	3 mo
Kohler 2013	67.7 (11)	67.9 (16.3)	65.9	43.75	72.72	72.91	36.36	25	59.09	56.25	59.09	47.91	33.14	14.58	31.81	18.75	3 mo	3 mo
Amiri-Nikpour 2015	65.23 (8.99)	66.52 (7.80)	46.15	48.14	69.23	77.78	17.39	29.62	46.15	40.74	34.61	37.03	19.23	18.51	34.61	29.62	3 mo	3 mo
Shamsaei 2017	69 (10.3)	68 (10.3)	44.44	38.89	50	55.56	50	16.67	27.8	33.33	33.33	22.22	NR	NR	NR	NR	3 mo	3 mo
Lu 2026	64.6 (11.1)	64.3 (10.4)	66.5	67.1	64.3	66.4	33.2	32.5	43.9	42.1	37.7	41.6	27.6	29.9	11	13.2	3 mo	3 mo

Data are presented as mean (SD) or percentage (%).

AIS = acute ischemic stroke, NR = Not Reported, TIA = transient ischemic attack.

### 3.2. Risk of bias, publication bias, and certainty of evidence assessment

Most included studies were judged to have a high overall risk of bias, mainly driven by concerns about deviations from intended interventions. Only 2 studies demonstrated low risk across all domains, while one study had some concerns (Fig. 2). No obvious funnel-plot asymmetry was observed; however, publication bias could not be reliably assessed because of the small number of studies contributing to each outcome (supplementary Figures 25 to 42, Supplemental Digital Content 27). Using the GRADE tool, the certainty of evidence was rated very low for the NIH stroke scale, BI, matrix metalloproteinase-9 (MMP-9) levels, and functional independence in terms of the mRS, while mortality, recurrent stroke/MI, and hemorrhagic conversion were rated low in terms of certainty of evidence. The complete GRADE assessment is given in Supplementary Table 2, Supplemental Digital Content 2.

**Figure 2.**
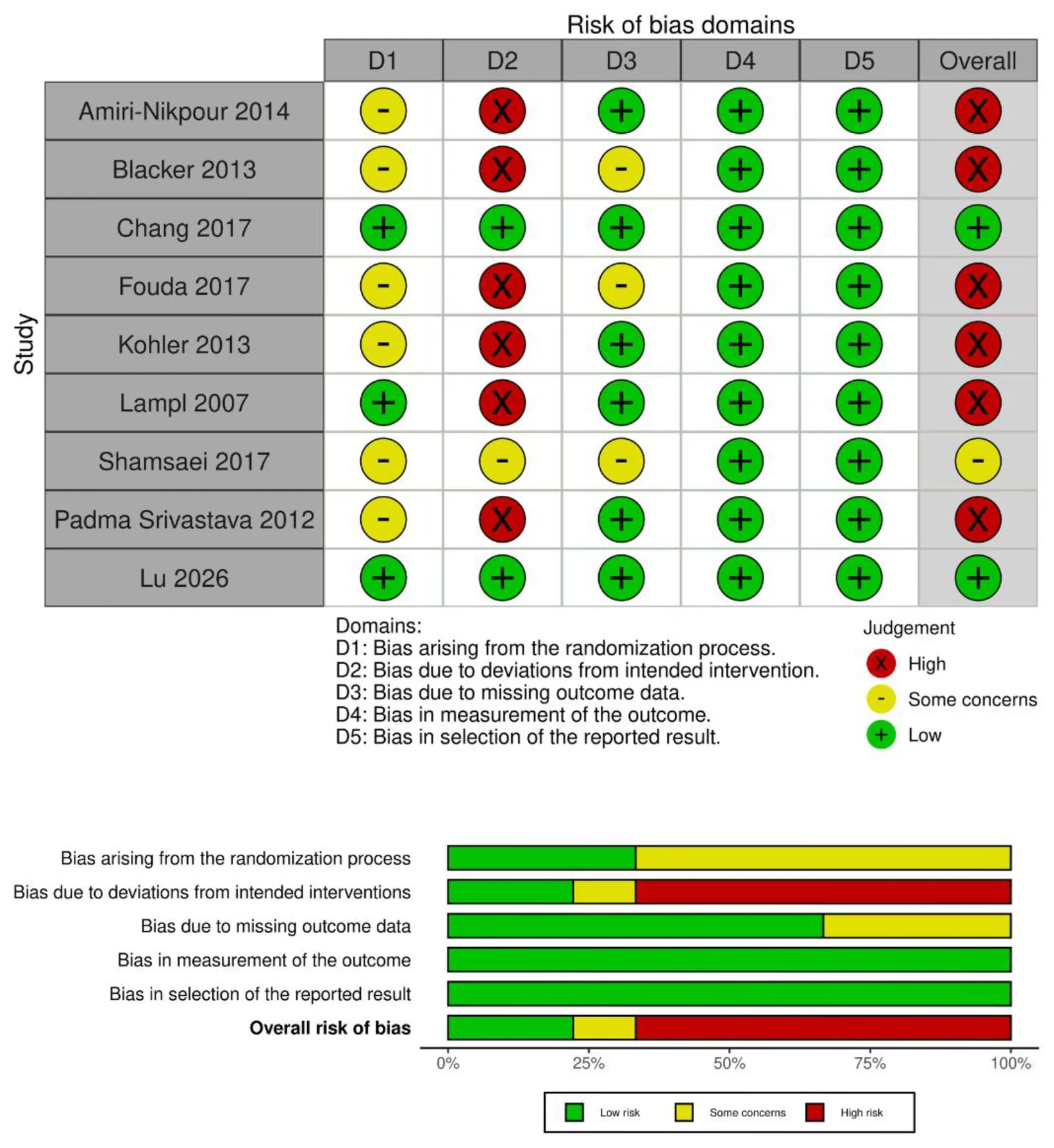
Quality Assessment of included RCT = randomised controlled trials.

### 3.3. Primary outcomes

#### 3.3.1. NIH stroke scale (NIHSS)

A total of 6 studies comprising 319 participants (154 in the minocycline group and 165 in the control group) were included in the 3-month NIHSS analysis. Overall, minocycline was associated with a reduction in NIHSS scores compared with control; however, this did not reach statistical significance (MD −1.78, 95% CI − 3.73–0.18; *P* = .074). Substantial heterogeneity was observed (*I^2^* = 90.3%).

Subgroup analysis revealed a differential treatment effect. In patients with AIS, minocycline significantly reduced NIHSS scores (MD − 2.43, 95% CI − 4.42 –− 0.44; *P* = .0169), although heterogeneity remained high (*I^2^* = 88.6%). In contrast, no significant benefit was observed in ICH patients (MD 0.70, 95% CI − 0.67–2.08; *P* = .3149), with no heterogeneity (*I^2^* = 0%). The interaction between subgroups was statistically significant (*P* = .0112), suggesting a potential variation in treatment effect based on stroke subtype (Fig. 3C).

**Figure 3.**
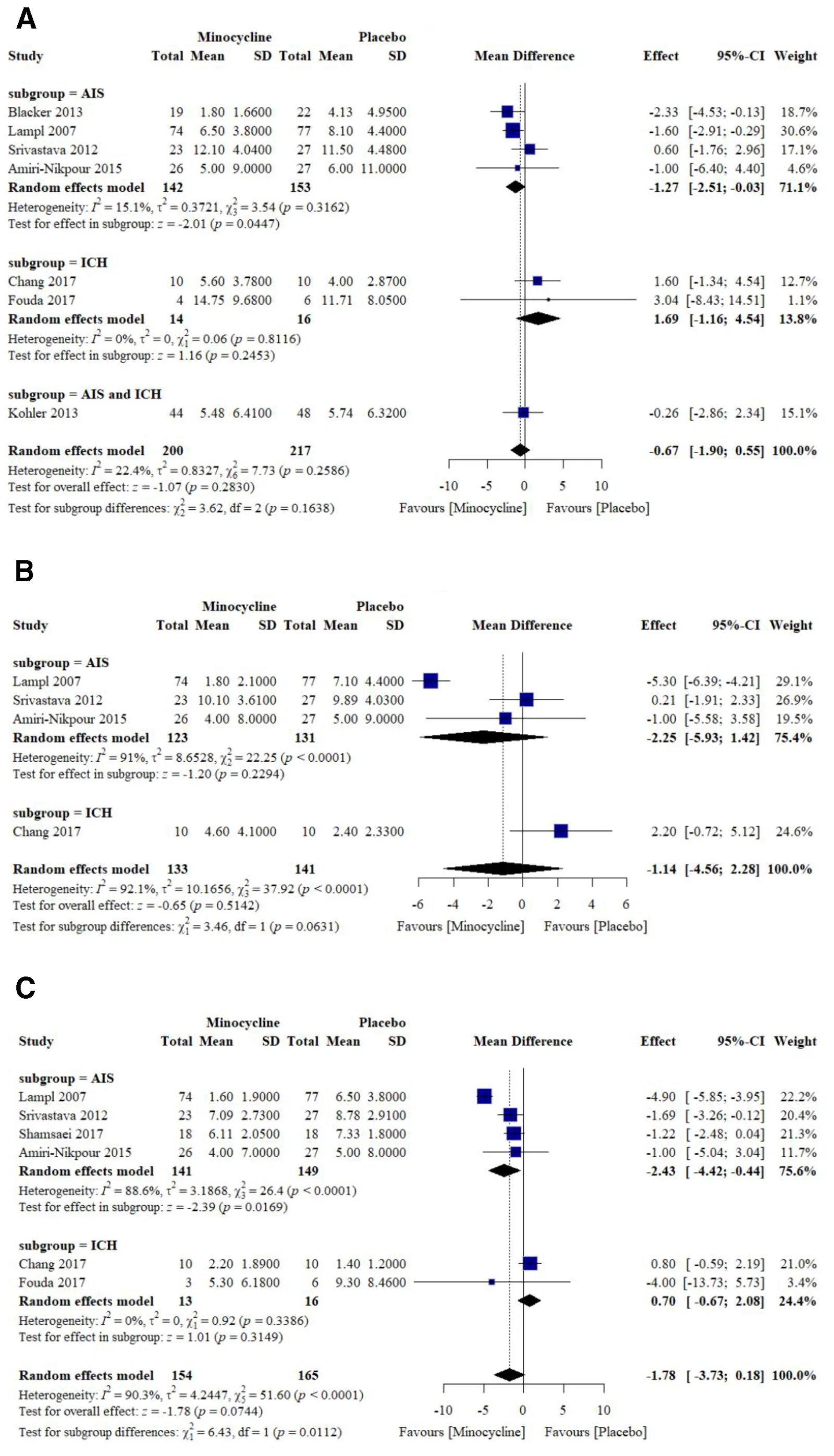
Forest Plots of (a) NIHSS at 5-7 days, (b) NIHSS at 30 days, (c) NIHSS at 3 months.

Sensitivity analysis using a leave-one-out approach demonstrated that exclusion of Lampl 2007 substantially reduced heterogeneity (*I^2^* = 44.0%) while maintaining a non-significant pooled estimate (MD −0.77, 95% CI − 2.06–0.52; *P* = .2435), indicating that the overall findings were robust despite heterogeneity. Omitting Chang 2017 resulted in the change in NIHSS at 3 months becoming significant in favor of minocycline (MD −2.49, 95% CI −4.40 – −0.58; *P* = .01). (Supplementary Figures 13-15, Supplemental Digital Content 15).

Short-term outcomes were consistent with the overall findings. At 5 to 7 days, there was no significant difference between groups (MD −0.67, 95% CI − 1.90 – 0.55; *P* = .283), although AIS patients showed a modest improvement (MD −1.27, 95% CI − 2.51–−0.03; *P* = .0447), which was not observed in ICH patients (Fig. 3A). Similarly, at 30 days, no significant difference was detected (MD −1.14, 95% CI −4.56–2.28; *P* = .514) (Fig. 3B).

#### 3.3.2. Functional independence (mRS 0–2)

Seven studies (n = 2071) were included in the analysis of mRS 0 to 2. The pooled estimate suggested a trend toward improved outcomes with minocycline, but this did not reach statistical significance (RR 1.25, 95% CI 1.00–1.55; *P* = .051), with high heterogeneity (*I^2^* = 75.5%) (Fig. 4A).

**Figure 4.**
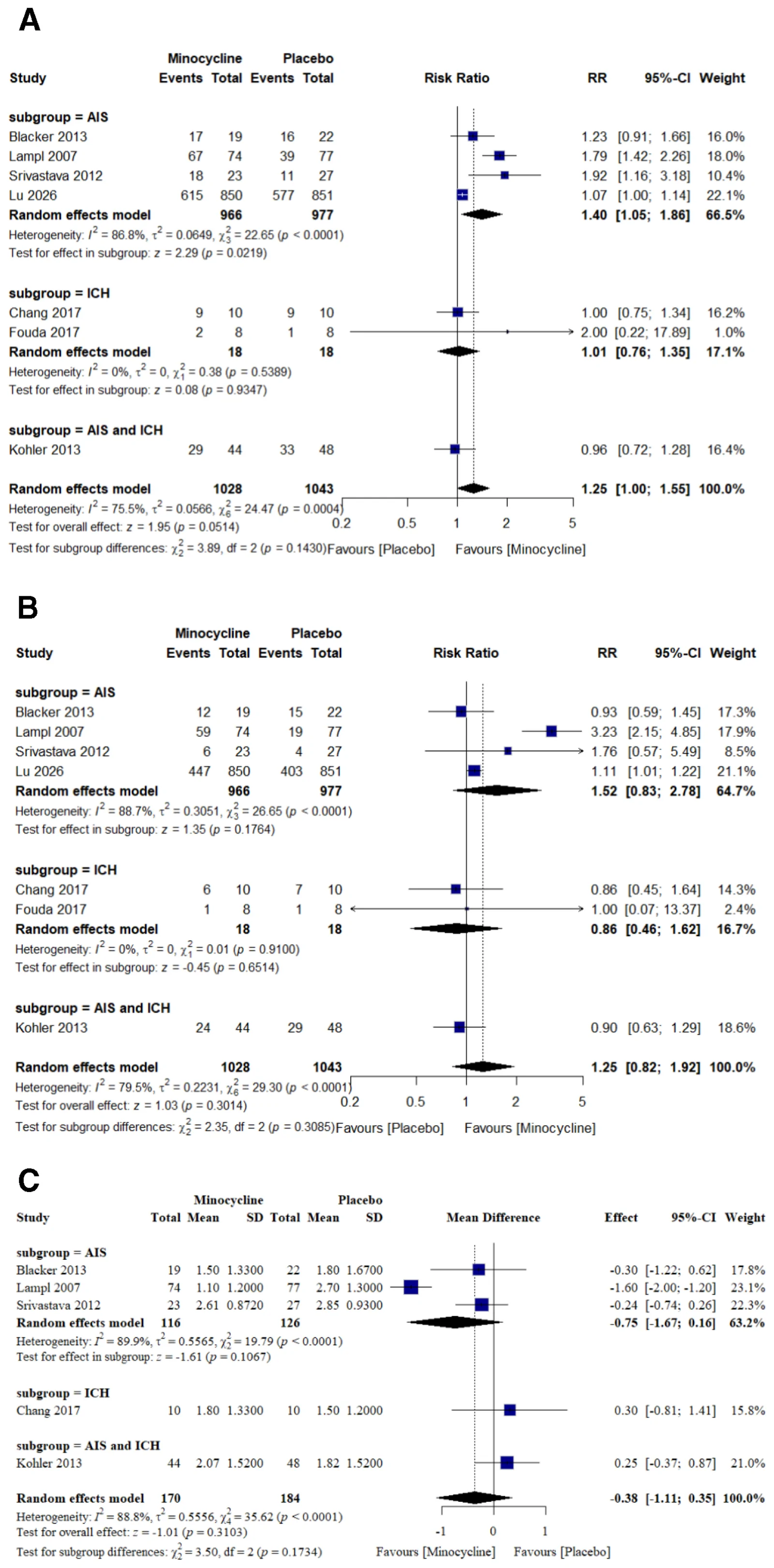
Forest Plots of (a) Functional independence (mRS 0–2) at 3 months, (b) Favorable functional outcome (mRS 0–1) at 3 months, (c) mRS at 30 days. CI = confidence interval, ICH = intracerebral hemorrhage, NIHSS = National Institutes of Health Stroke Scale.

In subgroup analysis, a statistically significant benefit was observed in AIS patients (RR 1.40, 95% CI 1.05–1.86; *P* = .0219), whereas no effect was seen in ICH patients (RR 1.01, 95% CI 0.76–1.35; *P* = .9347). However, because the test for subgroup interaction was not statistically significant (*P* = .1430), the AIS and ICH subgroup estimates should not be interpreted as evidence of a differential treatment effect.

Short-term mRS outcomes at 30 days (Fig. 4C) showed no significant differences between groups. Leave-one-out sensitivity analyses showed that statistical significance varied with the exclusion of individual trials; notably, exclusion of Lampl 2007 yielded a significant pooled effect with substantially reduced heterogeneity (RR 1.07, 95% CI 1.01–1.14; *P* = .0155). These findings indicate that the overall mRS 0 to 2 result was sensitive to individual-study exclusion and should therefore be interpreted cautiously. (Supplementary Figures 16-18, Supplemental Digital Content 18).

#### 3.3.3. Favorable functional outcome (mRS 0–1)

Seven studies, including 2071 participants (1028 in the minocycline group and 1043 in the control group), contributed to this analysis. There was no statistically significant difference between groups in achieving favorable functional outcomes (mRS 0–1) at 3 months (RR 1.25, 95% CI 0.82–1.92; *P* = .301). Considerable heterogeneity was observed (*I^2^* = 79.5%).

In subgroup analyses, neither AIS (RR 1.52, 95% CI 0.83–2.78; *P* = .176; *I^2^* = 88.7%) nor ICH (RR 0.86, 95% CI 0.46–1.62; *P* = .651; *I^2^* = 0%) populations demonstrated a statistically significant benefit. No significant interaction between subgroups was identified (*P* = .3085). (Fig. 4B).

Leave-one-out sensitivity analysis showed that exclusion of Lampl 2007 eliminated statistical heterogeneity (*I^2^* = 0%), while the pooled estimate remained non-significant (RR 1.07, 95% CI 0.95–1.20; *P* = .2658). (Supplementary Figures 19, Supplemental Digital Content 21).

#### 3.3.4. Barthel Index (BI)

Five studies, including 354 participants (170 minocycline and 184 control), were analyzed. Minocycline was associated with a higher BI at 3 months; however, this did not reach statistical significance (MD 6.99, 95% CI − 0.52–14.49; *P* = .0679). Heterogeneity was substantial (*I^2^* = 71.8%).

Subgroup analysis indicated a significant benefit in AIS (MD 12.34, 95% CI 5.45–19.23; *P* = .0004; *I^2^* = 47%), with no effect in ICH or mixed populations, and a significant subgroup difference (*P* = .0339). (Fig. 5C).

**Figure 5.**
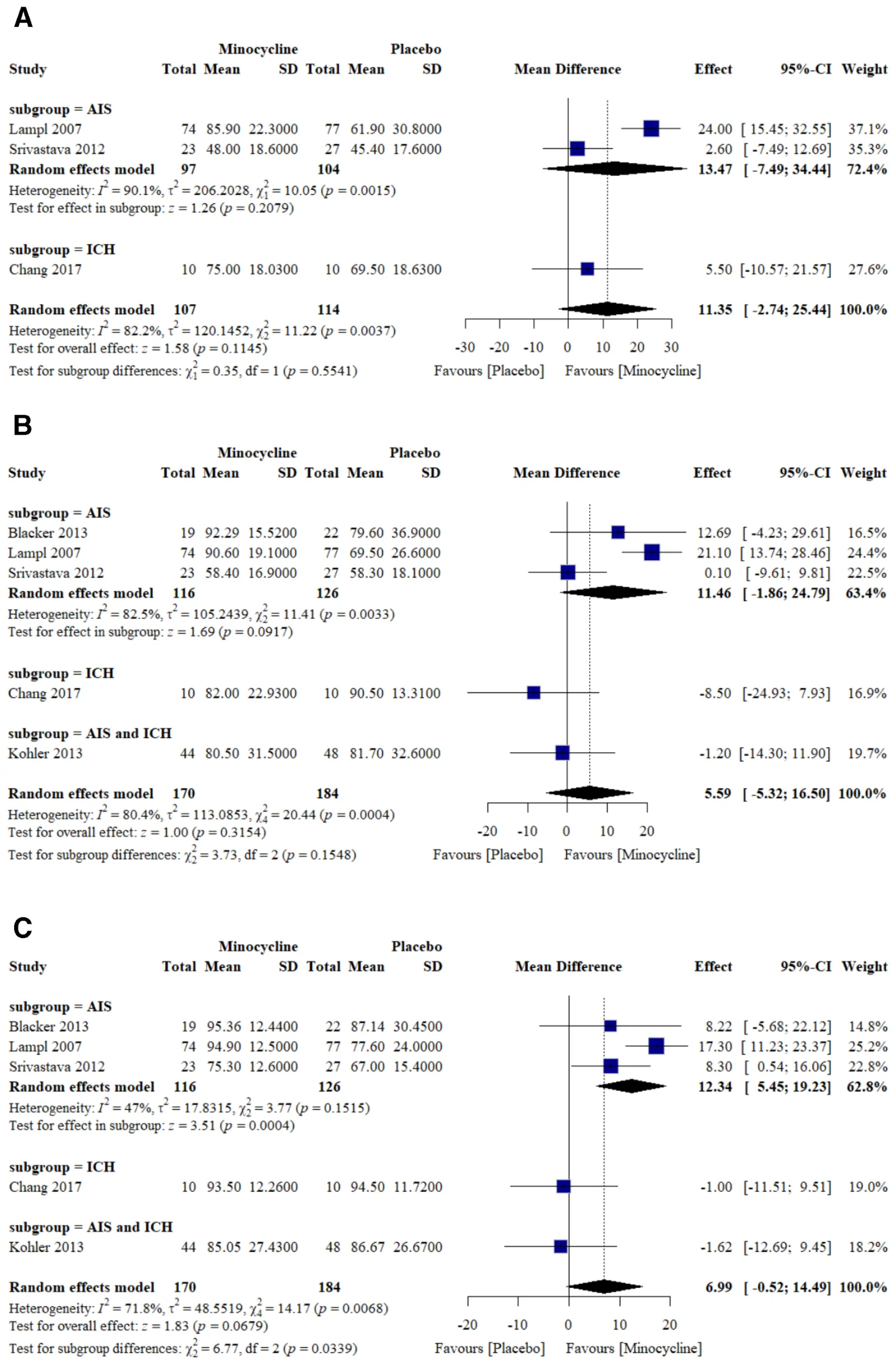
Forest Plots of (a) Barthel Index at 5-7 days, (b) Barthel Index at 30 days, (c) Barthel Index at 3 months. CI = confidence interval, ICH = intracerebral hemorrhage, SD = standard deviation.

Sensitivity analysis excluding Lampl 2007 reduced heterogeneity (*I^2^* = 11.5%) but did not change the overall conclusion (MD 3.82, *P* = .1933) (Supplementary Figures 20-22, Supplemental Digital Content 22).

Similarly, no significant differences were observed at earlier time points (5–7 days and 30 days) (Figure 5A and 5B).

#### 3.3.5. Mortality

Five studies involving 1976 participants (985 minocycline and 991 control) were included. Minocycline was associated with a numerically lower risk of mortality (RR 0.68, 95% CI 0.41–1.13), although this did not reach statistical significance (*P* = .135). Heterogeneity was negligible (*I^2^* = 0%) (Supplementary Figure 4, Supplemental Digital Content 6), and omitting any single study did not change the conclusion, confirming the result is robust (Supplementary Figure 24, Supplemental Digital Content 26).

### 3.4. Secondary outcomes

No significant differences were observed in the selected safety-related outcomes reported by the included trials; however, treatment-emergent adverse events were not reported consistently enough for a comprehensive pooled safety assessment.

No significant difference was observed in recurrent vascular events, including stroke or myocardial infarction (RR 0.86, 95% CI 0.48–1.53; *P* = .6079), with no heterogeneity (*I^2^* = 0%) (Supplementary Figure 1, Supplemental Digital Content 3), and omitting any single study did not change the conclusion, confirming the result is robust (Supplementary Figure 23, Supplemental Digital Content 25). Similarly, hemorrhagic transformation rates were comparable between groups (RR 0.84, 95% CI 0.13–5.32; *P* = .8497; *I^2^* = 22.8%) (Supplementary Figure 8, Supplemental Digital Content 10).

With respect to neuroimaging and biochemical outcomes, minocycline did not significantly influence ICH volume at either early (day 1–3) or later (day 5–11) time points (Supplementary Figures 5,6, Supplemental Digital Content 7). Likewise, 2 studies including 36 participants contributed to the MMP-9 analysis. The values entered in the meta-analysis were −88.00 ± 621.27 versus −116.00 ± 423.20 in Chang et al and 17.80 ± 69.11 versus −11.10 ± 106.92 in Fouda et al for the minocycline and control groups, respectively. The pooled effect was not statistically significant (MD 28.87, 95% CI − 57.81–115.55; *P* = .5139; *I^2^* = 0%). Given the very small sample size and differences in MMP-9 measurement methodology and assessment time points across studies, this finding should be considered exploratory (Supplementary Figures 2–3, and 7, Supplemental Digital Content 4).

### 3.5. Trial sequential analysis

In cases of mortality, the cumulative z-score did not cross the alpha-spending boundaries, nor did it cross the conventional naïve boundaries. Moreover, it was far from crossing the heterogeneity-adjusted required information size (HARIS), indicating that the analysis is underpowered to detect a significant difference in mortality (supplementary Figure 12, Supplemental Digital Content 14). Regarding improvement in NIHSS at 3 months, the z-score remained within the trial sequential monitoring boundaries at the current information size, suggesting that the observed improvement in neurological deficit is not yet statistically reliable, and further trials are needed to reach a definitive conclusion (supplementary Figure 9, Supplemental Digital Content 11). For mRS (0–2) at 3 months, the z-score failed to cross the alpha-spending boundary as well as the HARIS, indicating the current evidence is insufficient and underpowered to confirm a neurological benefit in terms of the mRS scale (supplementary Figure 10, Supplemental Digital Content 12). Similarly, the findings for the BI at 3 months indicate that the z-score, despite crossing the alpha-spending boundary early, crossed back into the zone of non-significance as more trials were added. The accrued information size reached 97% of HARIS at the final point of analysis. This suggests that current findings are not statistically reliable to reject the null hypothesis (supplementary Figure 11, Supplemental Digital Content 13).

## 4. Discussion

In our updated meta-analysis, we analyzed RCTs and found no statistically significant improvement in functional outcomes or survival rates after 3 months of minocycline treatment. However, we observed that minocycline treatment consistently produced positive results with special emphasis on neurological recovery. Although some AIS subgroup estimates were statistically significant, these findings should be considered exploratory given substantial heterogeneity and, for mRS 0 to 2, the absence of a statistically significant subgroup interaction. Our results also support existing research, which demonstrates that stroke patients display different treatment responses because their brain damage patterns vary.^[1,2]^

The pooled analysis of NIH Stroke Scale (NIHSS) scores at 3 months showed a numerical reduction in NIHSS scores that did not reach statistical significance. The AIS subgroup showed a significant treatment effect supporting the hypothesis that minocycline treats ischemic injury through its anti-inflammatory and neuroprotective actions.^[3–6]^ The primary injury mechanisms of ICH do not respond to treatment because they operate through mechanical disruption, hematoma expansion, and mass effect, which dominate the injury process.^[10,21]^ This shows how the stroke subtype acts as a key factor in predicting the response of patients to their respective treatments.

The functional outcomes followed a similar pattern. Our study found no substantial improvement in strict functional independence (mRS 0–1) but showed a trend toward improved mRS 0 to 2 scores with statistical significance in the AIS subgroup. The evidence suggests that minocycline enables people to achieve some functional improvement because it helps them recover partial independence while still reducing their total disability impact. In addition to this, the BI results showed a positive trend toward minocycline treatment but failed to show statistical significance because of 2 factors: small study populations and differences between research studies. The findings match previous clinical trials and meta-analyses, which showed small functional gains that occurred at inconsistent rates.^[4,8,21]^

Mortality was numerically lower with minocycline, but the difference was not statistically significant and should not be interpreted as evidence of a survival benefit. Previous early-phase trials and pooled analyses showed similar trends toward better survival outcomes, which did not achieve statistical significance.^[9,11]^

Our secondary outcomes showed no effect on the neurological progress of patients due to minocycline treatment during the first week and the subsequent month, showing that minocycline works through neuroprotection, which develops over time through its effects on inflammation, cell death, and brain healing processes.^[3,6]^ This delayed effect, shown by the drug action, targets secondary injury pathways rather than primary ischemic insult.

Despite compelling preclinical evidence, minocycline treatment did not show a significant effect on radiological and biochemical markers, including hematoma volume, perihematomal edema, or MMP-9 levels.^[5,21,22]^ This demonstrates a continuous translational obstacle that presents a challenge in stroke research.

The research shows that minocycline functions adequately with standard stroke treatments, which include thrombolysis, and previous studies have found that minocycline does not disrupt tissue-plasminogen activator fibrinolytic activity.^[8,22]^ No statistically significant differences were observed in the selected reported safety outcomes, including recurrent vascular events and hemorrhagic transformation; however, the available data do not permit a definitive assessment of overall treatment safety.

A key finding of our analysis was the substantial heterogeneity observed across several outcomes. Sensitivity analyses demonstrated that early-phase studies, together with individual trials, represented the most important factors creating this unpredictable outcome. Treatment effects experienced significant changes because patients received different doses at different times through different delivery and patient selection methods. Particularly, the delay at the start of therapy in some studies reduced the potential benefits of minocycline because neuroinflammation pathways become active during the initial stages of ischemic injury.^[3,4]^

The current research produces results that match some of the findings from past systematic reviews and meta-analyses, which showed that minocycline could improve neurological outcomes but failed to prove this link because the studies had small sample sizes and used fewer randomized trials. The present analysis delivers better evaluation results because it uses current data from large studies, which include multicenter randomized trials like EMPHASIS.^[11]^ Signals of potential benefit were observed mainly in AIS subgroups, whereas the limited ICH evidence did not demonstrate a statistically significant benefit.

The biological plausibility of these findings exists because minocycline demonstrates its mechanisms through microglial activation inhibition, pro-inflammatory cytokine suppression, matrix metalloproteinase activity reduction, and apoptosis attenuation.^[3,5,6]^ Scientists consider these processes to be essential for ischemia-reperfusion injury, which explains the reason for the different results between AIS and ICH.

Future trials may benefit from early prognostic stratification to identify patients at greater risk of neurological progression who could plausibly benefit from adjunctive neuroprotective therapy. Admission and serial NIHSS assessments, together with cardiac rhythm monitoring and ECG-derived markers, may provide complementary information regarding neurological severity and the likelihood of occult or subsequent atrial fibrillation. Emerging AI-based models integrating neurological assessments, ECG signals, imaging, laboratory findings, and clinical characteristics may further improve patient selection and individualized monitoring. Nevertheless, current evidence does not support the initiation of minocycline solely on the basis of disease severity.^[23–26]^

## 5. Strengths and limitations

Our study has multiple strengths. Firstly, we used RCTs, only including newly available evidence. Secondly, our study uses extensive subgroup and sensitivity tests to understand different factors that may have caused the results to differ. Lastly, we evaluated treatment outcomes with greater accuracy and used both clinical and radiological results as assessment methods.

However, our research has certain constraints that require Acknowledgments. Our study results showed significant heterogeneity across multiple outcomes that reflected variation in study design, treatment protocols, and patient populations. Some analyses were based on relatively smaller sample sizes, hence limiting statistical power. Furthermore, there were differences in dosing, timing, and routes of administration that may have further influenced the pooled estimates. Additionally, follow-up durations were shorter in several trials, and the data that were specific to ICH were limited, which prevents researchers from making definitive judgments about this specific patient group. The MMP-9 analysis was based on only 36 participants from 2 trials, and differences in assay methodology and measurement time points may introduce additional methodological heterogeneity; therefore, no firm conclusion regarding the effect of minocycline on MMP-9 can be drawn.

## 6. Conclusion

Minocycline did not significantly improve overall functional outcomes or reduce mortality after acute stroke. Although some subgroup analyses suggested potential neurological and functional benefits in AIS, these findings are exploratory and are limited by substantial heterogeneity and low or very-low certainty of evidence. TSA did not establish conclusive benefit. Larger randomized trials with standardized treatment timing, dosing, a, and outcome assessment are required to determine whether specific patient subgroups may benefit from minocycline.

## Author contributions

**Conceptualization:** Muhammad Shahzaib.

**Formal analysis:** Muhammad Shahzaib.

**Supervision:** Muhammad Shahzaib, Muhammad Roshaan.

**Methodology:** Muhammad Roshaan, Muhammad Noraiz Abbas Khan.

**Data curation:** Gaaitri Lohano, Muhammad Rehan Zahid.

**Writing – original draft:** Muhammad Roshaan, Gaaitri Lohano, Marium Fawad, Haram Nabeel.

**Writing – review & editing:** Muhammad Roshaan, Gaaitri Lohano, Haram Nabeel, Muhammad Noraiz Abbas Khan, Muhammad Rehan Zahid, Muhammad Nabeel, Nimra Afzal, Muhammad Shahryar, Eman Shahzad, Prince Kusimw’iragi.
























































































