# Supplementary material for: Minocycline in acute stroke: A systematic review and meta-analysis of randomized controlled trials and trial sequential analysis
Source: Medicine (Baltimore). 2026 Oct 23;105(43):e51287. doi: 10.1097/MD.0000000000051287 (PMC13706379; doi:10.1097/MD.0000000000051287)
Supplement: Supplementary file 9 [file medi-105-e51287-s011.pdf]

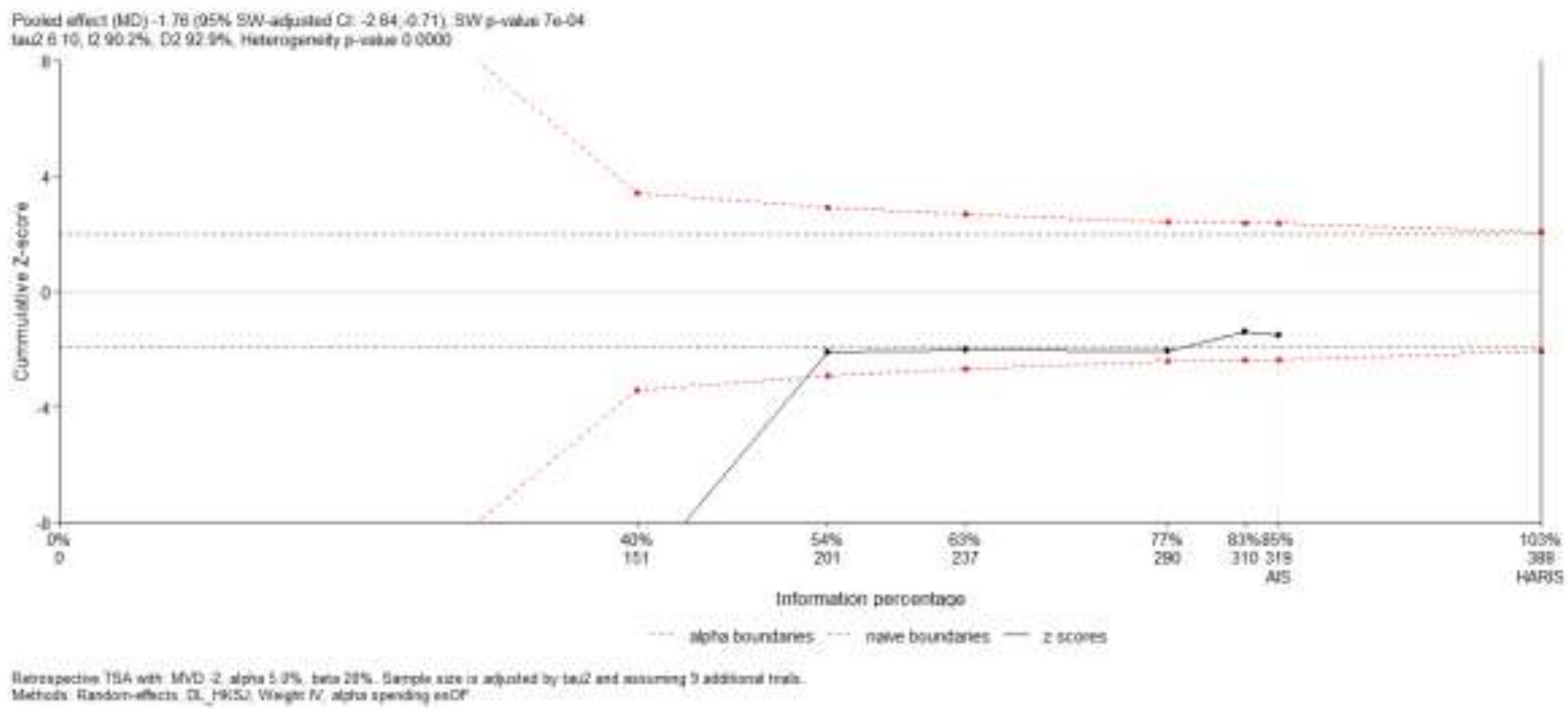

Supplementary figure 9. Trial sequential analysis for 3 month NIHSS
