# Supplementary material for: Minocycline in acute stroke: A systematic review and meta-analysis of randomized controlled trials and trial sequential analysis
Source: Medicine (Baltimore). 2026 Oct 23;105(43):e51287. doi: 10.1097/MD.0000000000051287 (PMC13706379; doi:10.1097/MD.0000000000051287)
Supplement: Supplementary file 13 [file medi-105-e51287-s015.pdf]

Study	MD	95%-CI	P-value	Tau2	Tau	I2
Omitting Blacker 2013	-0.28	[-1.65; 1.09]	0.6849	0.8624	0.9286	14.4%
Omitting Lampl 2007	-0.25	[-1.77; 1.27]	0.7462	1.0088	1.0044	14.1%
Omitting Srivastava 2012	-0.97	[-2.24; 0.29]	0.1324	0.6213	0.7882	15.0%
Omitting Amiri-Nikpour 2015	-0.63	[-1.94; 0.68]	0.3477	1.0054	1.0027	35.3%
Omitting Chang 2017	-1.10	[-2.16; -0.04]	0.0420	0.2275	0.4769	0%
Omitting Fouda 2017	-0.72	[-1.95; 0.52]	0.2546	0.8230	0.9072	31.2%
Omitting Kohler 2013	-0.68	[-2.16; 0.80]	0.3661	1.2779	1.1304	32.9%
**Random effects model**	**-0.67**	**[-1.90; 0.55]**	**0.2830**	**0.8327**	**0.9125**	**22.4%**

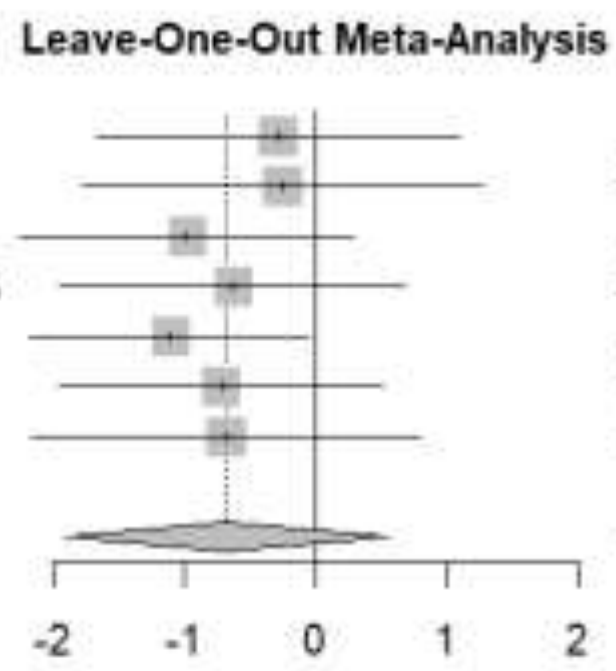

Supplementary figure 13. 5-7 day NIHSS Sensitivity analysis
