# Supplementary material for: Minocycline in acute stroke: A systematic review and meta-analysis of randomized controlled trials and trial sequential analysis
Source: Medicine (Baltimore). 2026 Oct 23;105(43):e51287. doi: 10.1097/MD.0000000000051287 (PMC13706379; doi:10.1097/MD.0000000000051287)
Supplement: Supplementary file 24 [file medi-105-e51287-s026.pdf]

Study	Leave-One-Out Meta-Analysis	RR	95%-CI	P-value	Tau2	Tau	I2
Omitting Blacker 2013		0.69	[0.41; 1.16]	0.1583	< 0.0001	0.0003	6.7%
Omitting Lampl 2007		0.79	[0.43; 1.46]	0.4551	< 0.0001	0.0024	0%
Omitting Lu 2026		0.70	[0.29; 1.70]	0.4315	0.1026	0.3204	10.3%
Omitting Fouda 2017		0.63	[0.38; 1.06]	0.0801	0	0	0%
Omitting Kohler 2013		0.65	[0.39; 1.11]	0.1154	< 0.0001	0.0006	3.4%
**Random effects model**		**0.68**	**[0.41; 1.13]**	**0.1354**	**< 0.0001**	**0.0023**	**0%**

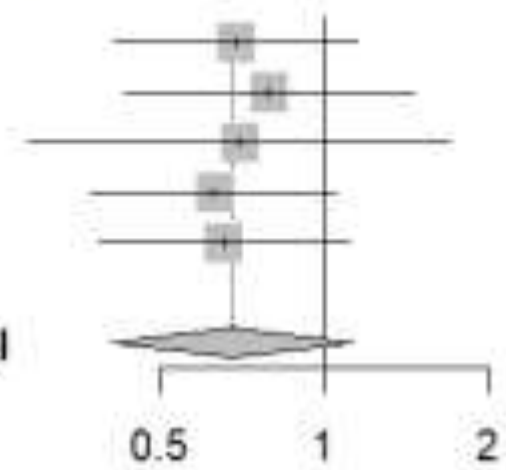

Supplementary figure 24. Mortality Sensitivity analysis
