# Supplementary material for: Minocycline in acute stroke: A systematic review and meta-analysis of randomized controlled trials and trial sequential analysis
Source: Medicine (Baltimore). 2026 Oct 23;105(43):e51287. doi: 10.1097/MD.0000000000051287 (PMC13706379; doi:10.1097/MD.0000000000051287)
Supplement: Supplementary file 43 [file medi-105-e51287-s001.docx]

Supplementary table 1. Search strategy

| **PubMed** | ("Stroke"[Mesh] OR "Ischemic Stroke"[Mesh] OR stroke OR "ischemic stroke" OR "cerebral infarction" OR "Cerebrovascular Accident" OR "acute stroke") AND ("Minocycline"[Mesh] OR minocycline OR minocin) |
| --- | --- |
| **Embase** | ('stroke'/exp OR 'ischemic stroke'/exp OR stroke:ti,ab,kw OR 'ischemic stroke':ti,ab,kw OR 'cerebral infarction':ti,ab,kw OR 'cerebrovascular accident':ti,ab,kw OR 'acute stroke':ti,ab,kw) AND ('minocycline'/exp OR minocycline:ti,ab,kw OR minocin:ti,ab,kw) |
| **Science direct** | ("stroke" OR "ischemic stroke" OR "cerebral infarction" OR "cerebrovascular accident" OR "acute stroke") AND ("minocycline" OR "minocin") |
| **Cochrane** | (stroke OR "ischemic stroke" OR "cerebral infarction" OR "cerebrovascular accident" OR "acute stroke"):ti,ab,kw  AND  (minocycline OR minocin):ti,ab,kw |
