# Supplementary material for: Minocycline in acute stroke: A systematic review and meta-analysis of randomized controlled trials and trial sequential analysis
Source: Medicine (Baltimore). 2026 Oct 23;105(43):e51287. doi: 10.1097/MD.0000000000051287 (PMC13706379; doi:10.1097/MD.0000000000051287)
Supplement: Supplementary file 44 [file medi-105-e51287-s002.docx]

Supplementary table 2. GRADE assessment

| **Certainty assessment** | | | | | | | **№ of patients** | | **Effect** | | **Certainty** | **Importance** |
| --- | --- | --- | --- | --- | --- | --- | --- | --- | --- | --- | --- | --- |
| **№ of studies** | **Study design** | **Risk of bias** | **Inconsistency** | **Indirectness** | **Imprecision** | **Other considerations** | **Minocycline** | **Control** | **Relative (95% CI)** | **Absolute (95% CI)** |  |  |
| **NIHSS at 3 months** | | | | | | | | | | | | |
| 6 | randomised trials | serious | very serious | not serious | serious | none | 154 | 165 | - | MD **1.78 lower** (3.73 lower to 0.18 higher) | ⨁◯◯◯ Very low |  |
| **modified Rankin Scale 0-1 at 3 months** | | | | | | | | | | | | |
| 7 | randomised trials | serious | very serious | not serious | serious | none | 555/1028 (54.0%) | 478/1043 (45.8%) | **RR 1.25** (0.82 to 1.92) | **115 more per 1,000** (from 82 fewer to 422 more) | ⨁◯◯◯ Very low |  |
| **modified Rankins Scale 0-2 at 3 months** | | | | | | | | | | | | |
| 7 | randomised trials | serious | very serious | not serious | serious | none | 757/1028 (73.6%) | 686/1043 (65.8%) | **RR 1.25** (1.00 to 1.55) | **164 more per 1,000** (from 0 fewer to 362 more) | ⨁◯◯◯ Very low |  |
| **Barthel Index at 3 months** | | | | | | | | | | | | |
| 5 | randomised trials | serious | serious | not serious | very serious | none | 170 | 184 | - | MD **6.99 higher** (0.52 lower to 14.49 higher) | ⨁◯◯◯ Very low |  |
| **Mortality** | | | | | | | | | | | | |
| 5 | randomised trials | serious | not serious | not serious | serious | none | 24/985 (2.4%) | 35/991 (3.5%) | **RR 0.68** (0.41 to 1.13) | **11 fewer per 1,000** (from 21 fewer to 5 more) | ⨁⨁◯◯ Low |  |
| **Recurrent stroke/ MI** | | | | | | | | | | | | |
| 4 | randomised trials | serious | not serious | not serious | serious | none | 56/994 (5.6%) | 58/998 (5.8%) | **RR 0.86** (0.48 to 1.53) | **8 fewer per 1,000** (from 30 fewer to 31 more) | ⨁⨁◯◯ Low |  |
| **Hemorrhagic conversion** | | | | | | | | | | | | |
| 2 | randomised trials | serious | not serious | not serious | serious | none | 3/115 (2.6%) | 4/121 (3.3%) | **RR 0.84** (0.13 to 5.32) | **5 fewer per 1,000** (from 29 fewer to 143 more) | ⨁⨁◯◯ Low |  |
| **ICH volume reduction** | | | | | | | | | | | | |
| 2 | randomised trials | serious | not serious | not serious | serious | none | 18 | 18 | - | MD **2.94 lower** (15.66 lower to 9.78 higher) | ⨁⨁◯◯ Low |  |
| **Perihematomal edema reduction** | | | | | | | | | | | | |
| 2 | randomised trials | serious | not serious | not serious | serious | none | 18 | 18 | - | MD **10.11 higher** (5.47 lower to 25.68 higher) | ⨁⨁◯◯ Low |  |
| **MMP-9 levels** | | | | | | | | | | | | |
| 2 | randomised trials | serious | not serious | serious | serious | none | 18 | 18 | - | MD **28.87 higher** (57.81 lower to 115.55 higher) | ⨁◯◯◯ Very low |  |

**CI:** confidence interval; **MD:** mean difference; **RR:** risk ratio
